# Binding the boundaries of chromatin domains

**DOI:** 10.1186/gb4183

**Published:** 2014-06-30

**Authors:** Vincenzo Pirrotta

**Affiliations:** 1 Department of Molecular Biology and Biochemistry, Rutgers University, 604 Allison Road, Piscataway, NJ 08854, USA

## Abstract

A new study proposes an integrated framework to improve our understanding of the multiple functions of insulator elements, and their architectural role in the genome.

See related research; http://genomebiology.com/2014/15/6/R82

## Insulator-binding proteins

Since their discovery in *Drosophila*, insulator elements and the proteins that bind to them have fascinated researchers for their unusual topology-dependent enhancer-blocking activities, and for the diverse roles they play in the genome. In recent years, attention has focused on their ability to promote interactions between different binding sites, producing higher-order, long-distance folding of chromatin. This could be the underlying feature common to the different roles that have been ascribed to them. A new article from the laboratory of Victor Corces now proposes an integrative framework to understand their function through their ability to determine the folding and architecture of genomic chromatin [1].

In *Drosophila*, five different insulator DNA-binding proteins have been described: Su(Hw), dCTCF, Zw5, BEAF32 and, in some contexts, GAGA-binding factor (GAF). The first three of these, like mammalian CTCF, are multi-zinc finger proteins with a similar structure. Two other proteins, CP190 and isoforms of Mod(mdg4), are known to interact with insulator-binding factors and are recruited to many, but not all, of their binding sites. CP190 and Mod(mdg4) contain a BTB/POZ domain known to mediate strong protein-protein interactions. At sites known to act as enhancer blocking insulators, they are essential for insulator function and are thought be the ‘glue’ that tends to hold together two or more insulator sites.

How insulators act as enhancer blockers remains mechanistically obscure, although several models have been proposed. They do not act as enhancer decoys that compete with promoters; instead they interfere with physical contact between chromatin elements on one side, and elements on the other side of their binding site. The best formulation so far, based largely on Su(Hw), was offered by Capelson and Corces [2], who proposed that insulator-binding complexes interact with one another through the ‘glue’ proteins CP190 and Mod(mdg4), organizing genomic chromatin into loops radiating from clusters of interacting insulator elements. The topology of these structures somehow prevents interactions between different chromatin loops.

Surprisingly, most of the *Drosophila* insulator proteins are phylogenetically recently derived. The exception is CTCF, the only insulator protein so far known in mammals. Homologs of the ‘glue’ proteins are not conserved in mammals. Instead, many but not all CTCF binding sites also bind cohesin. Cohesin and condensin are multi-protein ring-shaped complexes whose key structural constituents are called structural maintenance of chromosome (SMC) proteins. Cohesin and condensin can use their ring structure to hold together two chromatin fibers, and they serve important roles in chromosome condensation and segregation during mitosis. They are also important in chromatin architecture and gene expression during interphase. It has been shown that cohesin binding at CTCF binding sites depends on CTCF and that cohesin is required for CTCF insulator function. Thus, in some way, cohesin, like the ‘glue’ proteins in *Drosophila*, may gather together multiple CTCF binding sites.

## Genome architecture

Genomic mapping studies have revealed hundreds of binding sites for each of the insulator proteins in *Drosophila,* and more than 10,000 for mammalian CTCF. However, many of these do not have insulator function; instead, in many cases they are involved in folding the chromatin fiber, thus bringing appropriate regulatory regions together to contact promoters. Most insulator protein binding sites are invariant in different tissues or developmental stages, suggesting that they play structural roles in shaping the architecture of the genome. In the last few years, studies using Chromatin Conformation Capture (3C) technologies for genomic analysis of chromatin-chromatin interactions have revealed the presence of complex higher order organization in mammalian and *Drosophila* genomes. The genome is organized in topologically associated domains (TADs) within which chromatin-chromatin interactions are extensive, while interactions across domain borders are suppressed [3-8]. The domain borders therefore have the properties expected for insulator elements, which then become understandable in the broader context of genomic architecture. Many TAD borders in fact correspond to binding sites for insulator proteins. However, many more binding sites are found within domains, indicating that most do not act as either domain borders or insulators.

In a new study, Van Bortle *et al.*[1] considered first of all that insulator proteins function as organizers of genomic architecture rather than as local insulators, and they proposed to rename them architectural proteins (APs) and their binding sites as APBSs. They used *Drosophila* as a model because of its variety of known APs and they mapped an additional kind of element that has AP-like properties: TFIIIC, a DNA-binding complex that recruits RNA polymerase III (RNA Pol III) to targets such as tRNA genes. Clusters of tRNA genes in budding yeast and in mammalian genomes have insulator properties dependent on TFIIIC. Binding sites for TFIIIC unconnected with RNA Pol III promoters are also found in yeast, *Drosophila* and mammals, and are known as extra TFIIIC (ETC.) sites. The condensin complex is enriched at TFIIIC binding sites and is responsible for their spatial clustering in the nucleus. Thus, TFIIIC might constitute yet another AP, linked to the others by the association with SMC-containing complexes such as cohesin or condensin. Van Bortle *et al.* find that both of these SMC complexes are enriched at ETC. sites, and that ETC. sites and SMC complexes often coincide with sites that bind multiple APs. Thus, contrary to earlier conclusions, in *Drosophila*, as well as in mammals, APs are often associated with SMC complexes. In fact, the association is correlated with the degree of AP occupancy of a site: the more APs bound the more likely the association with SMC complexes. Furthermore, knocking down APs such as dCTCF leads to loss of cohesin at these sites.

## Combinatorial nature of boundaries

Armed with this collection of APs and their associated proteins (Table 1), Van Bortle *et al.* examined the relationship between their binding sites and the genomic architecture, as revealed by TADs [1]. Their key insight is that TAD boundaries and insulator function do not behave in an all-or-nothing fashion, but instead are combinatorial, and have graded functions. TAD boundaries correspond well to high occupancy APBSs, that is, sites where multiple APs bind within a short interval. This clustering distinguishes TAD boundaries from the scattered APBSs found within individual TADs. Furthermore, TAD boundaries vary in strength: some are weak, allowing significant interactions between sequences on either side, and others are strong, preventing interactions across the boundary region. The strength, measured by the ratio of interactions within TADs to interactions between flanking TADs, is highly correlated to the level of occupancy of APs at the TAD boundary. Van Bortle *et al.* calculated the level of occupancy only in terms of the number of different APs bound per unit length, not in terms of level of binding or number of binding sites for each AP. In other words, what they measured was the diversity of APs associated with a boundary rather than the level of occupancy. It is likely that the actual number of proteins bound would be important. Perhaps the most robust *Drosophila* insulator is the *gypsy* element isolated from the *gypsy* retrovirus, which consists of 12 binding sites for the Su(Hw) AP. In this case, the diversity is low but the cluster of binding sites produces a synergy that allows successful recruitment of the ‘glue’ and SMC components required for boundary/insulator function.

**Table 1:** Architectural proteins

***Drosophila *** **APs**	**Mouse ES cell APs**
dCTCF	CTCF
BEAF-32	
Su (Hw)	
CP190	
Mod (mdg4)	
DREF	
Chromator	
L(3)mbt	
dTFIIIC220	TFIIIC
Rad21 (cohesin)	Rad21 (cohesin
CAP-H2 (condensin II)	CAP-H2, CAP-D3 (condensin II)
	PRDM5

The architectural proteins used by Van Bortle *et al.* to characterize topologically associated domains (TADs) and their boundaries. ES, embryonic stem.

The discovery of the relationship between APBS clustering and the strength of TAD boundaries was made possible by the variety of different ABs known in *Drosophila*. This insight would not have been evident in mammals, where CTCF was the only insulator protein known. Going back to the extensive human and mouse Hi-C data and adding mapping data for TFIIIC, cohesin and condensin as well as CTCF and PRDM5 (another protein recently found to be frequently associated with the first four), Van Bortle *et al.* were able to confirm that the conclusions reached for *Drosophila* hold also for mammalian genomes.

## TAD boundaries and insulators

The properties of TAD boundaries are those expected of a good old-fashioned enhancer-blocking insulator. Might clustering of APBSs also explain which APBSs have insulator properties and which do not? Going back to *Drosophila,* APBSs that have been directly found to have insulator activity, Van Bortle *et al.* show that they are well correlated with the degree of occupancy of APs. Sites with robust insulator activity bind at least seven APs. Sites whose insulator activity is context-dependent bind, on average, five APs, while sites lacking demonstrable insulator activity bind an average of three and a half. Furthermore, insulators correspond, as expected, to TAD boundaries and the degree of insulator activity corresponds to the strength of the boundary.

**Figure 1:**
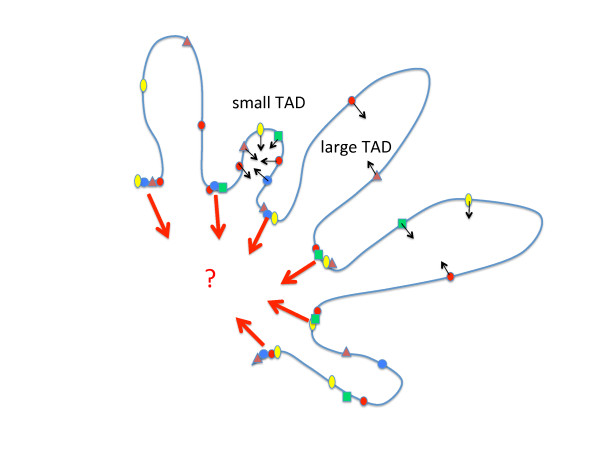
**Conjectured chromatin architecture.** The schematic drawing depicts TADs as chromatin loops bounded by regions of high architectural protein (AP) occupancy (multicolored objects), as proposed by Van Bortle *et al.*[1]. These are conjectured to interact with an unknown nuclear structure that provides topological constraints. The size of a TAD is determined by the density of interactions within the TAD. These are provided by APs, so that a relatively high density of AP binding sites results in a smaller TAD. Might the density of interactions provide a sufficient ‘gravitational’ pull to hold the chromatin of a TAD physically together and therefore in a preferentially interacting network? It is more difficult to explain how regions within a large TAD are prevented from interacting with regions in another large TAD. Patterns of gene activity or silencing and associated factors may also contribute to close the interaction horizon of TAD sequences.

This model brings together a multitude of observations in a satisfying framework. But it does not really explain how cohesin or the ‘glue’ proteins produce the barrier to interaction that generates the boundary/insulator. It is as if regions bracketed by APBS clusters tended to be segregated together in space, forming a package or TAD within which a higher frequency of interactions occurs at the expense of interactions with chromatin in other packages or TADs. Is there a greater physical distance between sequences in two adjacent TADs than between sequences in the same TAD, separated by the same length of DNA? If so, what accounts for this distance? Perhaps a clue is given by the observation that the density of APBSs in a given TAD is inversely correlated with the size of the TAD (Figure 1). Small TADs may therefore be created by the presence of numerous internal APBSs that promote interactions within the TAD at the expense of interactions between TADs. TAD boundaries might form when the local density of APBSs is so high that their interactions would all be preferentially local: between the APs binding at the boundary and at the exclusion of interactions with other APBSs. Alternatively, the high density of APBS at boundaries might make them interact preferentially with other high density sites. APs are found to form nuclear foci, so-called insulator bodies [9]. Perhaps such bodies are formed by the AP-mediated association of several boundary regions. How this would produce topological constraints to account for TADs and insulator function is not clear. The key may be the role of the SMC complexes that are enriched at high occupancy sites. How cohesin functions in regulating the segregation of sister chromatids during cell division has been well dissected. Much less clear is how it functions at its widespread binding sites during interphase. Clearly, much remains to be explained but the work of Van Bortle *et al.* has provided us with some important insights.

## Abbreviations

AP: Architectural protein; APBS: Architectural protein binding site; ETC: Extra TFIIIC (not associated with RNA Pol III promoters); RNA Pol III: RNA polymerase III; SMC: Structural maintenance of chromosome; TAD: Topologically associated domain.

## Competing interests

The author declares that he has no competing interests.
